# Development of a nomogram to predict the prognosis of patients with secondary bone tumors in the intensive care unit: a retrospective analysis based on the MIMIC IV database

**DOI:** 10.1007/s00432-024-05667-9

**Published:** 2024-03-28

**Authors:** Weikang Li, Jinliang Li, Jinkui Cai

**Affiliations:** Department of Orthopedics, Wuhan Third Hospital, Tongren Hospital of Wuhan University, Wuhan, 430074 China

**Keywords:** Secondary bone tumors, MIMIC database, Nomogram, Prediction tool, Cancer survival

## Abstract

**Purpose:**

The present study aimed to develop a nomogram to predict the prognosis of patients with secondary bone tumors in the intensive care unit to facilitate risk stratification and treatment planning.

**Methods:**

We used the MIMIC IV 2.0 (the Medical Information Mart for Intensive Care IV) to retrieve patients with secondary bone tumors as a study cohort. To evaluate the predictive ability of each characteristic on patient mortality, stepwise Cox regression was used to screen variables, and the selected variables were included in the final Cox proportional hazard model. Finally, the performance of the model was tested using the decision curve, calibration curve, and receiver operating characteristic (ROC) curve.

**Results:**

A total of 1028 patients were enrolled after excluding cases with missing information. In the training cohort, albumin, APSIII (Acute Physiology Score III), chemotherapy, lactate, chloride, hepatic metastases, respiratory failure, SAPSII (Simplified Acute Physiology Score II), and total protein were identified as independent risk factors for patient death and then incorporated into the final model. The model showed good and robust prediction performance.

**Conclusion:**

We developed a nomogram prognostic model for patients with secondary bone tumors in the intensive care unit, which provides effective survival prediction information.

## Introduction

Bone is one of the most common sites of metastasis for malignant tumors, affecting many patients with advanced cancer (Coleman et al. 2020a). Bone metastases often lead to skeletal morbidity called skeletal-related events (SREs) (Moos et al. 2019). In general, SREs reduce overall survival and are associated with loss of mobility and social function, decreased quality of life, and substantial increase in medical costs (Coleman et al. 2020b). In most cases, the treatment of bone metastases focuses on preventing disease progression and alleviating symptoms. And within the context of multidisciplinary supportive care, years of disease control and reduction of the impact of metastatic bone disease on physical function can be achieved (Coleman 2006). Cancer patients require ICU (intensive care unit) admission after cancer progression, surgery, radiotherapy-related complications, or complications from severe acute illness (Soares et al. 2010). Patients with bone metastases are more severely ill and more likely to have complications than cancer patients without bone metastases, and an increased need for medical care (Fornetti et al. 2018; Jimenez-Andrade et al. 2010). Therefore, it is important to identify high-risk patients with poor prognosis in the intensive care unit. It helps clinicians to improve treatment strategies in time to improve the prognosis of patients.

Currently, multiple studies have explored the prognostic factors and established models to predict the prognosis of patients with various types of malignant tumors (Baba et al. 2018; Vichapat et al. 2011; Fang et al. 2020; Gurney et al. 2013; Liu et al. 2016; Mao et al. 2018). Other studies have developed models to predict bone metastasis in patients with malignant tumors (Teng et al. 2020; Ellmann et al. 2019; Hou et al. 2021). Bone metastases are common in patients with malignant tumors, whereas few studies have been conducted with bone metastases as research subjects to explore the prognosis of patients (Guo et al. 2008; Abdelazeem et al. 2022).

The nomogram has been widely used as a predictive method for the prognosis of patients with various diseases (Park 2018; Lv et al. 2021; Hess 2021; Yuan and Wu 2021), and its visual interface allows accurate quantification of the risk of independent risk factors by score. Clinicians can calculate scores from the characteristics on the column line graphs to predict the probability of death or illness of a patient. In this study, a nomogram prognostic model based on Cox proportional hazard model was established by employing a large multicenter database MIMIC IV 2.0 as the data source, and patients with secondary bone tumors in the intensive care unit as the research subjects. The aim was to explore the independent risk factors affecting the prognosis of patients and to facilitate clinicians to identify high-risk patients for more accurate clinical decision-making.

## Methods

### Study cohort and data

Data were extracted from the MIMIC IV 2.0 database on patients diagnosed with secondary bone tumors according to the International Classification of Diseases codes, Ninth Revision (198.5) and Tenth Revision (C7B.03, C79.5). To improve usability, we have collected routine, readily accessible clinical indicators. The collected data included patient demographics (age, gender, ethnicity), body mass index, comorbidities (cancers, acute kidney injury, hepatic metastases, pulmonary metastasis, brain metastases, acidosis, respiratory failure, heart failure, atrial fibrillation, hypertension), treatment information (chemotherapy, parenteral nutrition, radiotherapy, mechanical ventilation), laboratory results (hematology: atypical lymphocytes, metamyelocytes, mean corpuscular hemoglobin concentration, mean corpuscular volume, mean hemoglobin content; biochemical test: pO_2_, calculated total CO_2_, pCO_2_, pH, base excess, lactate, free calcium; biochemical test: glutamic-pyruvic transaminase, alkaline phosphatase, glutamic oxaloacetic transaminase, creatinine kinase MB, albumin, total protein, anion gap, bicarbonate, calcium, creatinine, chloride, potassium), and prognosis scores(APSIII, SOFA, SAPSII), with cases with missing data excluded. For patients with multiple ICU admissions, we selected data from the first ICU admission of the patient for analysis. In addition, we used data from patients within 24 h of admission to the ICU for the analysis. If the patient had multiple measurements within 24 h of admission to the ICU, the data from the first measurement were used.

### Statistical analysis

Each variable was divided into training and validation data sets, with the categorical variables described by percentage (%), non-normally distributed continuous variables expressed using median and quartiles, and normally distributed continuous variables described using mean and standard error [mean (S.E.)]. The chi-square test was adopted to compare differences in categorical variables, and the t-test or Mann–Whitney U test was used to compare differences between two groups of continuous variables. The starting point for follow-up was defined as the time the patient was admitted to the ICU. The primary outcome indicator for this study was the long-term mortality of the patients. Date of death is extracted from two sources: the hospital information system and the Massachusetts State Registry of Vital Records and Statistics. For the training cohort, feature selection was performed using univariate Cox regression and stepwise Cox regression based on AIC (Akaike Information Criterion) with both selections. Variables with *P* < 0.05 in the univariate analysis were included in the stepwise Cox regression, while variables with *P* < 0.05 in the stepwise Cox regression were included in the final Cox proportional hazard model, and the corresponding nomogram was generated. The multicollinearity of the variables in the model was detected by calculating the variance inflation factor (VIF), and a VIF higher than 2 was considered to have multicollinearity among the variables. Overall survival at 1 month, 3 months, 1 year, and 3 years was estimated using the nomograms. The discrimination ability of the model was evaluated by the area under the time-dependent receiver operating characteristic curve (time-dependent AUC). The calibration graph was used to assess the agreement between the predicted and actual values of the model. The *survival* package (version 3.5-7) was used for univariate Cox regression and stepwise Cox regression, the *rms* package (version 6.7-0) was used for plotting nomogram and calibration curves, the *survivalROC* package (version 1.0.3.1) was used for plotting ROC curves, and the *dcurves* package (version 0.4.0.9) was used for plotting decision curves. All statistical analyses were performed using R 4.2.1., with a bilateral *P*-value < 0.05 considered statistically significant.

## Results

### Study cohort

A total of 1357 patients with bone metastases admitted to the ICU were identified from the database, and after excluding patients with missing information (*N* = 329), a total of 1028 patients were finally included in the study (median survival time: 642.50 days). Including 720 in the training cohort (median survival time: 624.00 days) and 308 in the validation cohort (median survival time: 695.50 days) (Table 1).

**Table 1:** Description of all characteristics

Factors		Overall	Training set	Validation set	*P*-value
N		1028	720	308	
	Survival time (Days)	642.50 [94.00, 2385.00]	624.00 [96.75, 2259.25]	695.50 [80.75, 2562.75]	0.641
	Status = Dead (%)	325 (31.6)	229 (31.8)	96 (31.2)	0.898
	Gender = Male (%)	583 (56.7)	402 (55.8)	181 (58.8)	0.423
	Age (Years)	65.00 [56.00, 74.00]	66.00 [56.00, 75.00]	64.00 [54.75, 73.00]	0.234
	Ethnicity (%)				0.415
	Asian	54 (5.3)	41 (5.7)	13 (4.2)	
	Black	123 (12.0)	91 (12.6)	32 (10.4)	
	Other	114 (11.1)	75 (10.4)	39 (12.7)	
	White	737 (71.7)	513 (71.2)	224 (72.7)	
	BMI (kg/m^2^)	27.80 [24.40, 31.60]	27.67 [24.40, 31.67]	28.00 [24.48, 31.05]	0.972
	Type of cancer (%)				0.165
	Respiratory system	144 (14.0)	101 (14.0)	43 (14.0)	
	Digestive system	75 (7.3)	47 (6.5)	28 (9.1)	
	Reproductive organ of the male	57 (5.5)	39 (5.4)	18 (5.8)	
	Urinary system	48 (4.7)	38 (5.3)	10 (3.2)	
	Breast	35 (3.4)	27 (3.8)	8 (2.6)	
	Lymphatic and hematopoietic systems	21 (2.0)	19 (2.6)	2 (0.6)	
	Skin and soft tissue	15 (1.5)	10 (1.4)	5 (1.6)	
	Reproductive organ of the female	5 (0.5)	4 (0.6)	1 (0.3)	
	Ill-defined, unspecified sites	509 (49.5)	361 (50.1)	148 (48.1)	
	Other	119 (11.6)	74 (10.3)	45 (14.6)	
Treatment	Mechanical ventilation = Yes (%)	92 (8.9)	64 (8.9)	28 (9.1)	1
	Radiotherapy = Yes (%)	79 (7.7)	58 (8.1)	21 (6.8)	0.579
	Parenteral nutrition = Yes (%)	88 (8.6)	67 (9.3)	21 (6.8)	0.236
	Chemotherapy = Yes (%)	389 (37.8)	273 (37.9)	116 (37.7)	0.995
Complications	AKI = Yes (%)	485 (47.2)	340 (47.2)	145 (47.1)	1
	Hepatic metastases = Yes (%)	384 (37.4)	260 (36.1)	124 (40.3)	0.234
	Pulmonary metastasis = Yes (%)	266 (25.9)	179 (24.9)	87 (28.2)	0.29
	Brain metastases = Yes (%)	230 (22.4)	167 (23.2)	63 (20.5)	0.377
	Acidosis = Yes (%)	288 (28.0)	202 (28.1)	86 (27.9)	1
	RF = Yes (%)	365 (35.5)	252 (35.0)	113 (36.7)	0.655
	Heart failure = Yes (%)	221 (21.5)	155 (21.5)	66 (21.4)	1
Blood count	MCH (%)	29.90 [28.20, 31.50]	29.90 [28.20, 31.50]	30.00 [28.30, 31.50]	0.701
	MCHC (%)	33.30 [32.30, 34.20]	33.30 [32.30, 34.20]	33.35 [32.38, 34.20]	0.723
	MCV (%)	90.00 [85.00, 94.00]	90.00 [85.00, 93.00]	89.50 [85.00, 94.00]	0.789
	Atypical lymphocytes (%)	0.26 [0.00, 0.91]	0.24 [0.00, 0.83]	0.35 [0.00, 1.00]	0.097
	Bands (%)	1.90 [0.00, 3.67]	1.83 [0.00, 3.64]	2.00 [0.00, 3.72]	0.758
	Metamyelocytes (%)	0.44 [0.00, 1.02]	0.46 [0.00, 1.00]	0.39 [0.00, 1.02]	0.918
Biochemical	ALT				0.418
	< 40 U/L	859 (83.6)	596 (82.8)	263 (85.4)	
	40–120 U/L	135 (13.1)	97 (13.5)	38 (12.3)	
	121–400 U/L	23 (2.2)	17 (2.4)	6 (1.9)	
	> 400 U/L	11 (1.1)	10 (1.4)	1 (0.3)	
	ALP				0.79
	< 40 U/L	15 (1.5)	12 (1.7)	3 (1.0)	
	40–100 U/L	597 (58.1)	414 (57.5)	183 (59.4)	
	101–400 U/L	375 (36.5)	266 (36.9)	109 (35.4)	
	> 400 U/L	41 (4.0)	28 (3.9)	13 (4.2)	
	AST				0.135
	$\le$ 40 U/L	891 (86.7)	632 (87.8)	259 (84.1)	
	> 40 U/L	137 (13.3)	88 (12.2)	49 (15.9)	
	Creatinine (mg/dL)	0.90 [0.70, 1.20]	0.90 [0.70, 1.20]	0.90 [0.70, 1.20]	0.962
	Potassium (mmol/L)	4.09 [3.87, 4.30]	4.10 [3.88, 4.30]	4.06 [3.84, 4.26]	0.182
	Creatinine kinase MB (ng/mL)	4.00 [2.46, 7.00]	4.00 [2.00, 6.85]	4.00 [3.00, 7.00]	0.149
	Albumin (g/dL)	3.80 [3.30, 4.20]	3.80 [3.30, 4.20]	3.80 [3.27, 4.30]	0.848
	Total protein (g/dL)	6.51 [6.18, 6.93]	6.52 [6.18, 6.97]	6.50 [6.18, 6.89]	0.65
	Calcium (mg/dL)	9.10 [8.60, 9.60]	9.10 [8.60, 9.60]	9.20 [8.60, 9.51]	0.831
	Chloride (mmol/L)	102.00 [99.00, 104.00]	102.00 [99.00, 104.00]	102.00 [99.00, 104.00]	0.17
Blood gas	pO_2_ > 80 (%)	71 (6.9)	58 (8.1)	13 (4.2)	0.037
	pCO_2_ (mmHg)	40.00 [35.00, 44.89]	40.00 [35.00, 45.00]	41.00 [36.00, 44.26]	0.363
	Base excess (mmol/L)	0.00 [-3.00, 1.00]	0.00 [-3.02, 1.00]	− 0.16 [− 3.00, 1.00]	0.8
	Calculated total CO_2_ (mEq/L)	25.00 [22.10, 28.00]	25.00 [22.00, 28.00]	25.00 [23.00, 27.00]	0.945
	Free calcium (mmol/L)	1.12 [1.10, 1.16]	1.13 [1.10, 1.16]	1.12 [1.10, 1.16]	0.942
	Lactate (mmol/L)	1.61 [1.30, 1.96]	1.60 [1.30, 1.97]	1.62 [1.30, 1.95]	0.554
	pH	7.38 [7.35, 7.43]	7.38 [7.35, 7.43]	7.38 [7.35, 7.43]	0.484
	Anion gap (mmol/L)	15.00 [13.00, 17.00]	15.00 [13.00, 17.00]	15.00 [13.00, 17.00]	0.428
	Bicarbonate (mmol/L)	26.00 [24.00, 28.00]	26.00 [24.00, 28.00]	26.00 [24.00, 28.00]	0.875
Scores	APSIII	41.00 [31.00, 53.00]	40.00 [31.00, 53.00]	41.50 [32.00, 53.00]	0.438
	SOFA	3.00 [2.00, 6.00]	3.00 [2.00, 6.00]	3.00 [2.00, 6.00]	0.359
	SAPSII	41.00 [33.00, 48.00]	41.00 [33.00, 48.00]	40.00 [32.00, 49.00]	0.734

*AKI* acute kidney injury, *RF* respiratory failure, *ALT* glutamic-pyruvic transaminase, *ALP* alkaline phosphatase, *MCV* mean corpuscular volume, *HF* heart failure, *AF* atrial fibrillation, *AST* aspartate aminotransferase, *Creatinine kinase MB* muscle and brain fraction of creatinine kinase, *MCH* mean corpuscular hemoglobin, *MCHC* medium corpuscular hemoglobin concentration, *BMI* body mass index, *APSIII* Acute Physiology Score III, *SOFA* Sequential Organ Failure Assessment, *SAPSII* Simplified Acute Physiology Score II

### Feature selection and model building

**Fig. 1:**
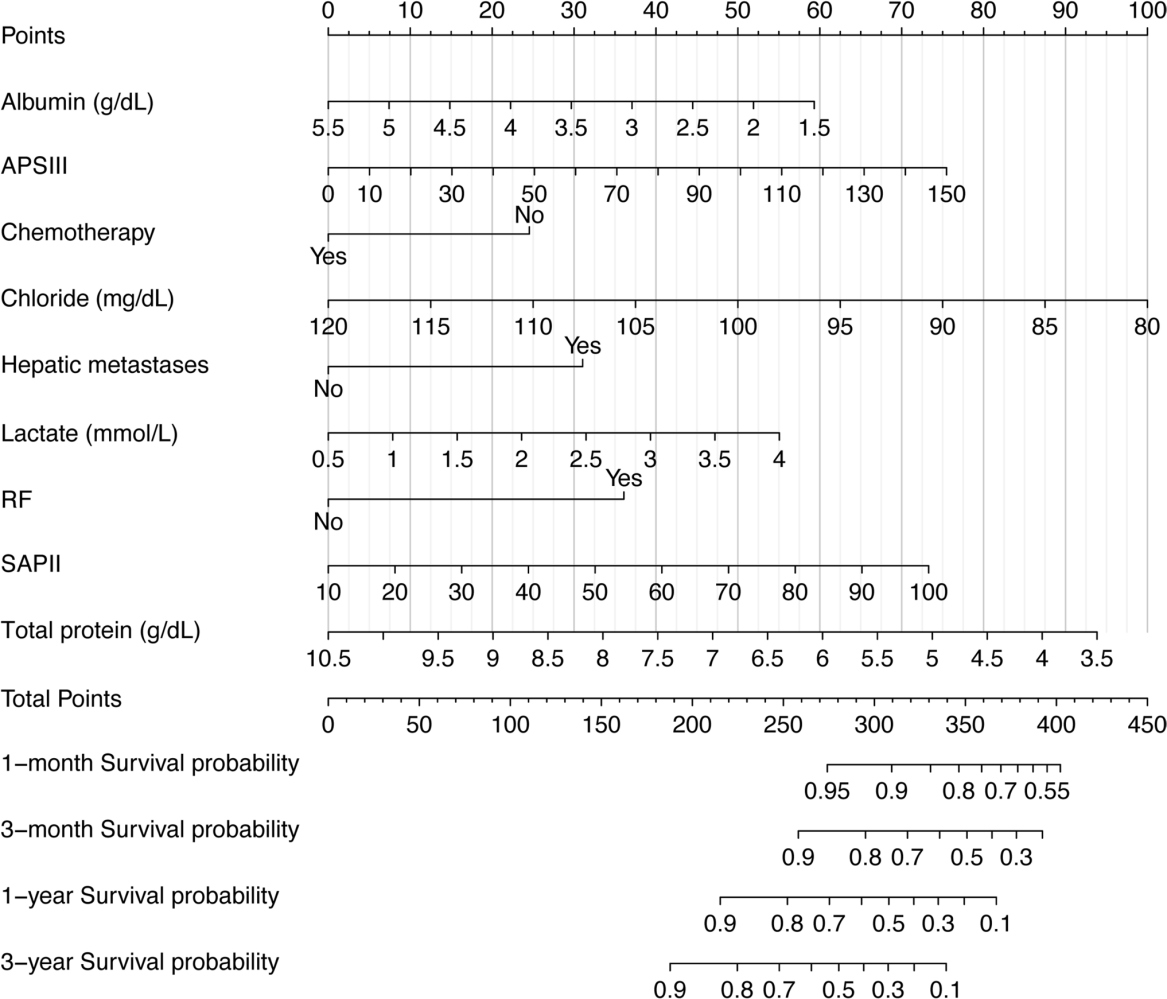
The nomogram for predicting survival in patients with secondary bone tumors. *RF* respiratory failure, *APSIII* Acute Physiology Score III, *SAPSII* Simplified Acute Physiology Score II

**Fig. 2:**
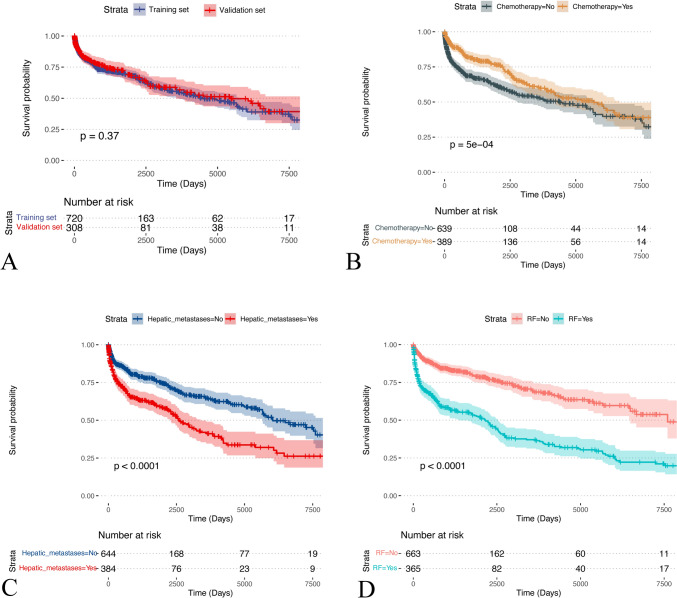
**A** KM curves grouped by training and validation sets. **B**–**D** Kaplan–Meier curves plotted by subgroups Chemotherapy, Hepatic metastases, and RF. RF, respiratory failure

Feature selection by univariate Cox regression and stepwise Cox regression showed that nine features, including albumin, APSIII, chemotherapy, lactate, chloride, hepatic metastases, respiratory failure, SAPSIII, total protein, were independent predictors of prognosis in patients with secondary bone tumors in the intensive care unit (Table 2). The VIF of the variables in the model was calculated and the results were all below 2 (albumin: 1.166, APSIII: 1.705, chemotherapy: 1.107, chloride: 1.080, hepatic metastases: 1.084, lactate: 1.129, respiratory failure: 1.079, SAPSII: 1.733, total protein: 1.181), showing no multicollinearity. The Cox proportional hazard model was established based on the above characteristics, and the nomogram was drawn as shown in Fig. 1. In the nomogram, the total score (Total Points) for each patient is calculated by adding the scores corresponding to each feature (Points), and the total score corresponds vertically to the scale on the predictor (1-month, 3-month, 1-year, and 3-year survival probability), i.e., the patient’s survival probability. If a patient’s ultimate total score (Total Points) is 300, then the patient’s probability of survival at 1 month, 3 months, 1 year, and 3 years is 90–95%, 80%, 60%, and 40%, respectively. In addition, for the categorical variables included in the model, we plotted Kaplan–Meier curves according to their grouping (Fig. 2).

**Table 2:** The results of the feature selection

Factors		Levels	Univariate analysis		Multivariate analysis	
			HR (95% CI)	*P*-value	HR (95% CI)	*P*-value
Demography	Age (Years)		1.01 (1.00–1.02)	0.0628		
	Gender	Female (Reference)				
		Male	1.10 (0.85–1.43)	0.4718		
	Ethnicity	Asian (Reference)				
		Black	0.79 (0.45–1.37)	0.4017		
		Other	0.69 (0.37–1.29)	0.2412		
		White	0.68 (0.42–1.1)	0.1136		
	BMI (kg/m^2^)		1.00 (1.00–1.00)	0.9116		
	Cancers	Breast (Reference)				
		Digestive system	2.09 (0.78–5.64)	0.1438		
		Reproductive organ of the female	0 (0–Inf)	0.9926		
		Ill-defined, unspecified sites	1.96 (0.80–4.79)	0.1403		
		Lymphatic and hematopoietic systems	1.27 (0.40–4.03)	0.6818		
		Other	1.49 (0.55–4.03)	0.4353		
		Reproductive organ of the male	1.32 (0.44–3.94)	0.6195		
		Respiratory system	2.14 (0.84–5.47)	0.1127		
		Skin and soft tissue	0.45 (0.05–3.84)	0.4648		
		Urinary system	0.96 (0.31–2.96)	0.9503		
Complications	Acidosis	No (Reference)				
		Yes	0.82 (0.62–1.08)	0.1581		
	AKI	No (Reference)				
		Yes	0.96 (0.74–1.25)	0.7829		
	HF	No (Reference)				
		Yes	0.75 (0.55–1.04)	0.084		
	RF	No (Reference)				
		Yes	2.85 (2.18–3.72)	< 0.0001*	2.08 (1.58–2.75)	< 0.0001*
	Pulmonary metastasis	No (Reference)				
		Yes	1.08 (0.81–1.45)	0.5838		
	Hepatic metastases	No (Reference)				
		Yes	1.76 (1.36–2.29)	< 0.0001*	1.88 (1.43–2.48)	< 0.0001*
	Brain metastases	No (Reference)				
		Yes	1.30 (0.96–1.75)	0.0902		
Treatments	Chemotherapy	No (Reference)				
		Yes	0.61 (0.47–0.80)	0.0003*	0.61 (0.46–0.81)	0.0006*
	Radiotherapy	No (Reference)				
		Yes	0.86 (0.53–1.39)	0.5389		
	Mechanical ventilation	No (Reference)				
		Yes	1.36 (0.94–1.97)	0.103		
	Parenteral nutrition	No (Reference)				
		Yes	0.96 (0.62–1.48)	0.8446		
Blood count	MCH (%)		0.99 (0.95–1.04)	0.7346		
	MCHC (%)		0.90 (0.83–0.98)	0.0112		
	MCV (%)		1.01 (0.99–1.02)	0.4553		
	Metamyelocytes (%)		1.10 (1.02–1.18)	0.0096		
	Bands (%)		1.03 (1.00–1.05)	0.018		
	Atypical lymphocytes (%)		0.95 (0.86–1.05)	0.334		
Biochemical	Albumin (g/dL)		0.57 (0.47–0.68)	< 0.0001*	0.74 (0.61–0.9)	0.0032*
	ALP (U/L)	< 40	—			
		40–100	1.15 (0.43, 3.12)	0.8		
		101–400	1.96 (0.73, 5.31)	0.2		
		> 400	5.34 (1.86, 15.4)	0.002		
	ALT (U/L)	< 40 (Reference)				
		40–120	1.18 (0.87, 1.62)	0.3		
		121–400	1.92 (1.12, 3.29)	0.017		
		> 400	1.46 (0.54, 3.91)	0.5		
	AST (U/L)	$\le$ 40 (Reference)				
		> 40	1.28 (0.90–1.83)	0.1755		
	Creatinine (mg/dL)		1.03 (0.99–1.08)	0.1763		
	Creatinine kinase MB (ng/mL)		1.00 (0.99–1.00)	0.6714		
	Potassium (mmol/L)		1.05 (0.83–1.34)	0.6801		
	Total protein (g/dL)		0.67 (0.58–0.79)	< 0.0001*	0.76 (0.64–0.91)	0.0028*
	Calcium (mg/dL)		0.98 (0.83–1.16)	0.8104		
	Chloride (mmol/L)		0.93 (0.91–0.96)	< 0.0001*	0.95 (0.92–0.98)	0.0008*
Blood gas	Anion gap (mmol/L)		1.08 (1.04–1.12)	0.0002*		
	Base excess (mmol/L)		0.98 (0.96–1.01)	0.1342		
	Bicarbonate (mmol/L)		0.96 (0.92–1.00)	0.0314		
	Calculated total CO2 (mEq/L)		0.99 (0.96–1.02)	0.5118		
	Free calcium (mmol/L)		4.96 (1.18–20.93)	0.0292		
	Lactate (mmol/L)		1.52 (1.26–1.83)	< 0.0001*	1.38 (1.14–1.67)	0.0011*
	pCO_2_ (mmHg)		0.99 (0.97–1.01)	0.3055		
	pO_2_ (mmHg)	< 80 (Reference)				
		$\ge$ 80	1.25 (0.83–1.89)	0.2796		
	pH		0.82 (0.09–7.94)	0.8656		
Scores	SAPSII		1.04 (1.03–1.04)	< 0.0001*	1.02 (1.01–1.03)	0.0017*
	APSIII		1.02 (1.02–1.03)	< 0.0001*	1.01 (1–1.02)	0.0030*
	SOFA		1.15 (1.11–1.18)	< 0.0001*		

*P-value < 0.05

*AKI* acute kidney injury, *RF* respiratory failure, *ALT* glutamic-pyruvic transaminase, *ALP* alkaline phosphatase, *MCV* mean corpuscular volume, *HF* heart failure, *AF* atrial fibrillation, *AST* aspartate aminotransferase, *Creatinine kinase MB* muscle and brain fraction of creatinine kinase, *MCH* mean corpuscular hemoglobin, *MCHC* medium corpuscular hemoglobin concentration, *BMI* body mass index, *APSIII* Acute Physiology Score III, *SOFA* Sequential Organ Failure Assessment, *SAPSIII* Simplified Acute Physiology Score II

### Validation of the model

**Fig. 3:**
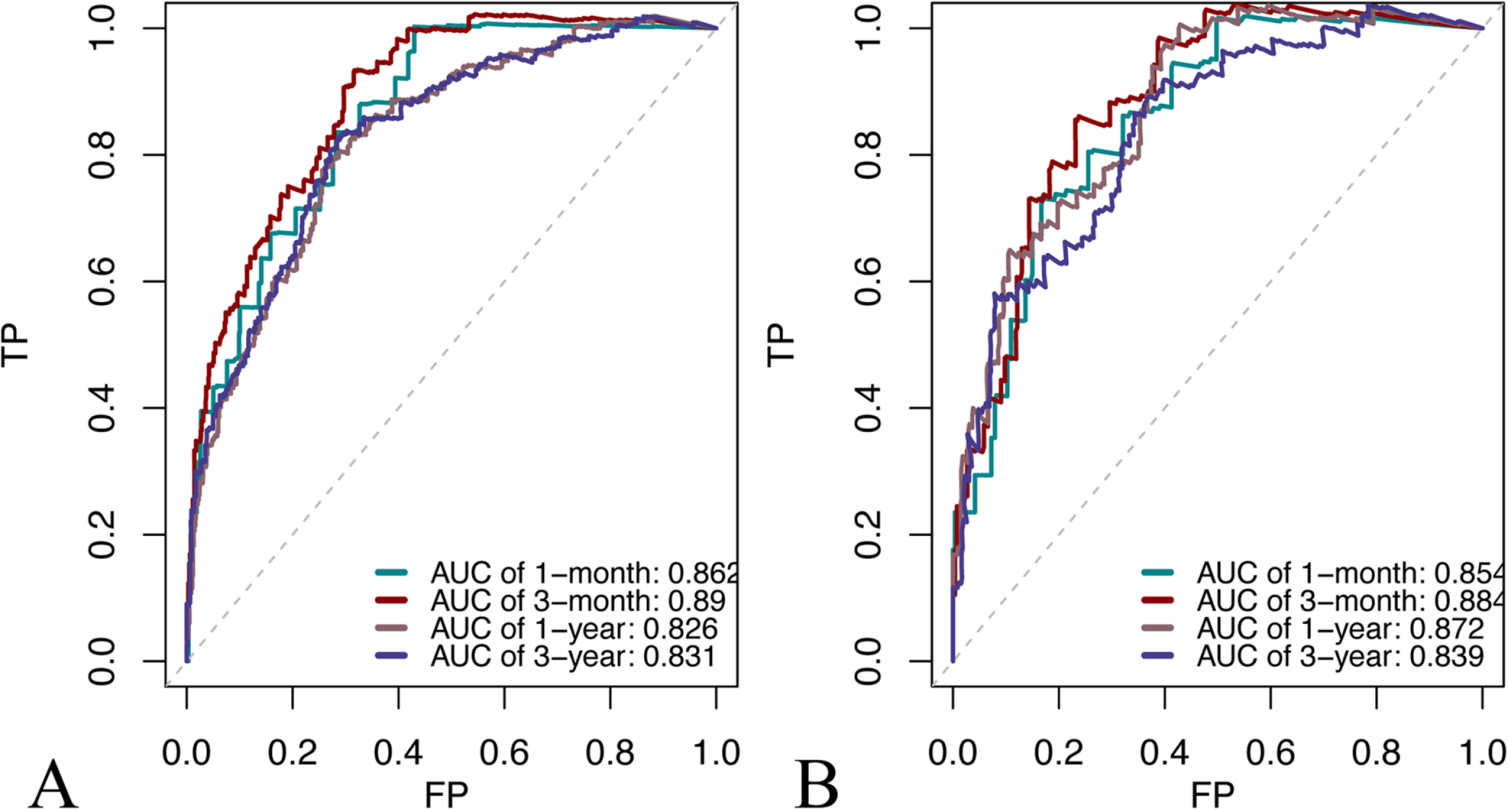
Time‐dependent AUC of using the nomogram to predict overall survival (OS) probability within 1 month, 3 months, 1 year, and 3 years in the training cohort (**A**) and validation cohorts (**B**)

**Fig. 4:**
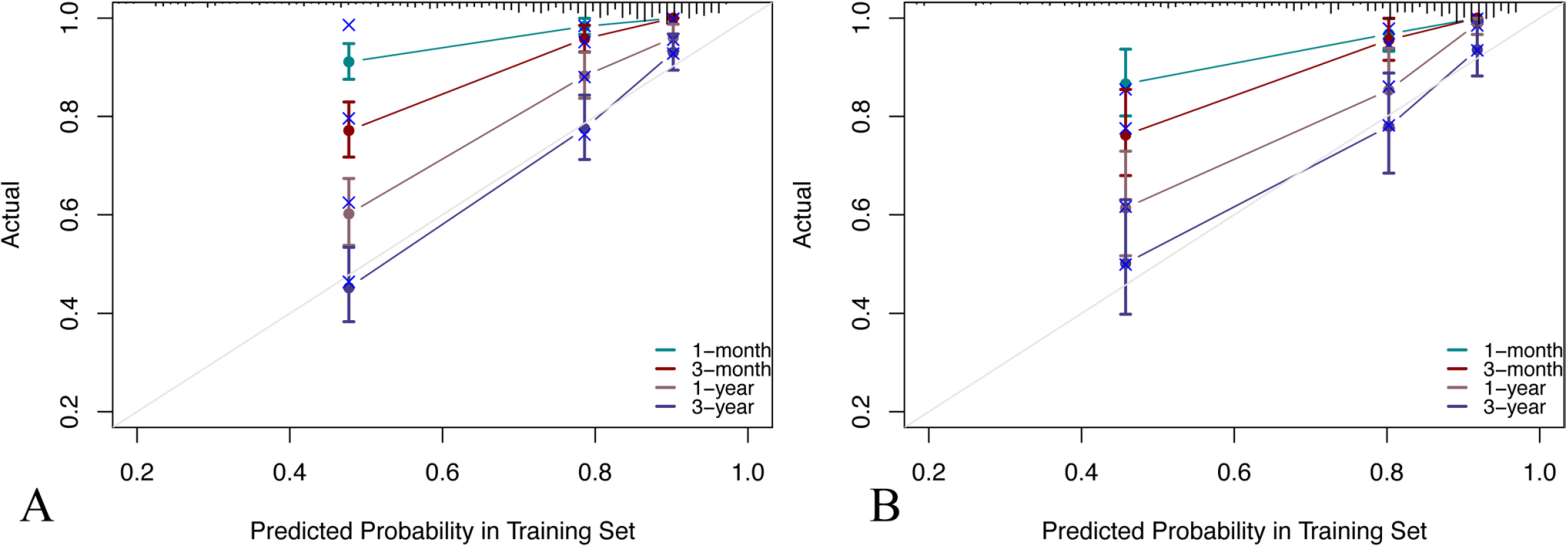
Calibration curves of the nomogram: **A** Calibration curves of 1-month, 3-month, 1-year, and 3-year OS for patients in the training cohort. **B** Calibration curves of 1-month, 3-month, 1-year, and 3-year OS for patients in the validation cohort

**Fig. 5:**
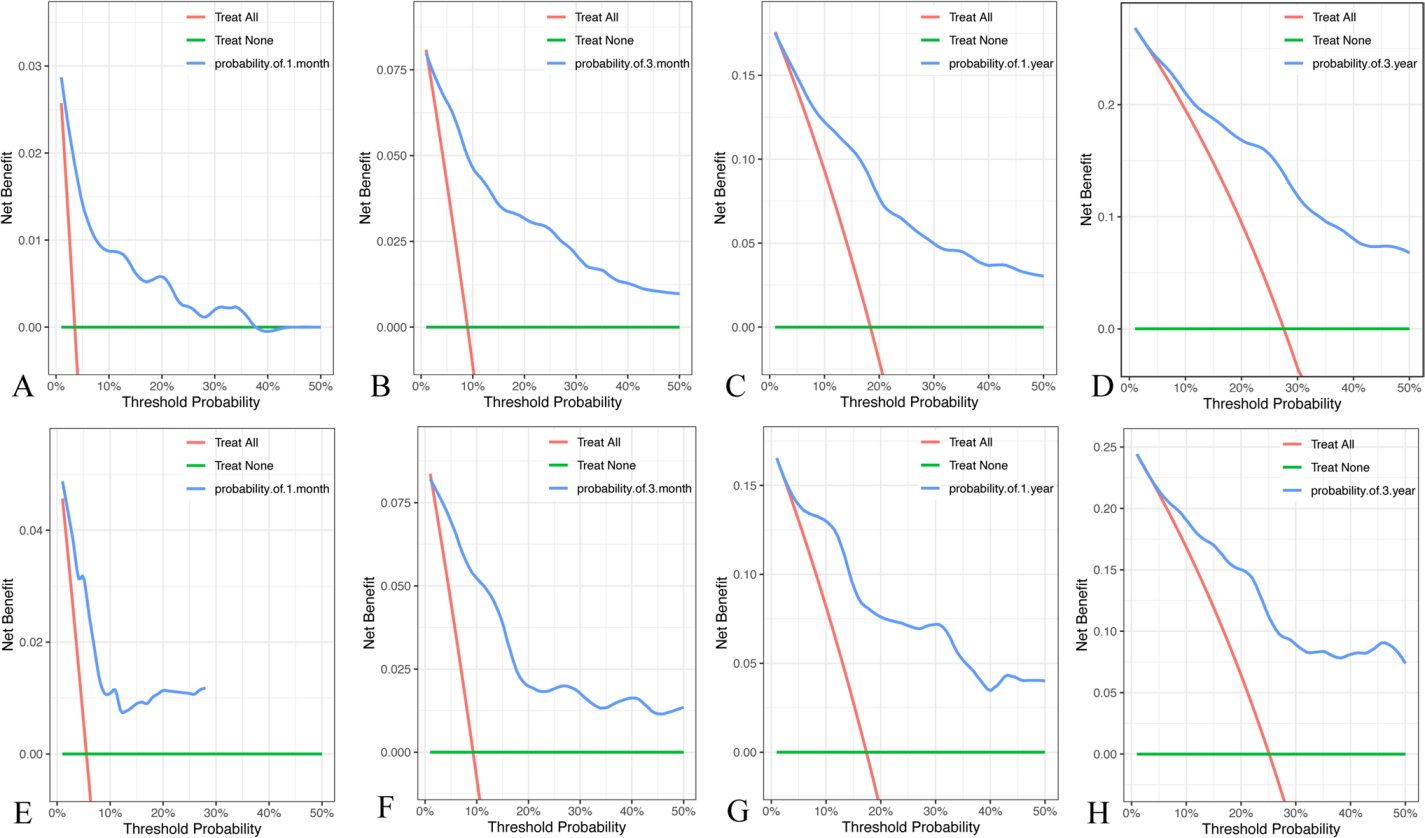
Decision curve analysis of the nomogram: **A** 1‐month survival benefit in the training cohort. **B** 3‐month survival benefit in the training cohort. **C** 1‐year survival benefit in the training cohort. **D** 3‐year survival benefit in the training cohort. **E** 1‐month survival benefit in the training cohort. **F** 3‐month survival benefit in the training cohort. **G** 1‐year survival benefit in the training cohort. **H** 3‐year survival benefit in the training cohort

The ROC curve, calibration curve, and decision curve were plotted to validate the model. The results of the ROC curve analysis showed that the AUC of the nomogram model for predicting the mortality in the training cohort at 1 month, 3 months, 1 year, and 3 years was 0.862, 0.890, 0.826, and 0.831, respectively; the AUC of the for predicting model for predicting the mortality in the validation cohort at 1 month, 3 months, 1 year, 3 years was 0.854, 0.884, 0.872, and 0.839, respectively (Fig. 3). And the model exhibited good predictive accuracy. The calibration curve analysis revealed that the agreement between the predicted and the actual values was within an acceptable range (Fig. 4). In addition, we plotted decision curves (Fig. 5). The green horizontal line in the figure shows the benefit if none of the patients received the intervention, the red bias line shows the benefit if all the patients received the intervention, and the blue curve shows the benefit if they received the intervention as judged by the model. The figure shows that our model has a large net gain in both the training and validation cohorts.

## Discussion

In the present study, we studied patients with secondary bone tumors in the intensive care unit and developed a nomogram model to predict patient prognosis based on patient demographic information, laboratory test indicators, and comorbidities/surgical history. The model achieved an AUC of above 0.8 in both the training and validation cohorts, showing good predictive value.

Most current studies on secondary bone tumors have focused on bone metastases from specific tumors (Li et al. 2021; Lang et al. 2013; Huang et al. 2019; Sun et al. 2019), and few pan-cancer studies have been conducted on bone metastases in all cancer patients. However, there is a certain commonality in patients who develop secondary bone tumors, especially in patients with bone metastases admitted to the intensive care unit. An earlier similar study analyzed prognostic factors based on 216 patients with bone metastases (Teshima et al. 1990), but the study cohort was not limited to the intensive care unit. Independent predictors of prognosis in patients with bone metastases in the intensive care unit remain uncertain. Hence, we developed a predictive model that can predict the prognosis of patients with secondary bone tumors in the intensive care unit to provide supporting data for future studies.

Our model showed that nine characteristics, including low albumin, APSIII, chemotherapy, high lactate, low chloride, hepatic metastases, respiratory failure, SAPSIII, and low total protein, were independent predictors of prognosis in patients with secondary bone tumors in the intensive care unit. Among them, albumin, chemotherapy, chloride, and total protein were shown to be protective factors; while, APSIII, hepatic metastases, SAPSIII, SOFA, and lactate were promoting factors of mortality. Among the protective biomarkers, total protein and albumin are often used as indicators of nutritional status and hepatic synthetic function (Hülshoff et al. 2013), and exogenous albumin is frequently treated as a nutritional support drug in critically ill patients (Farrugia 2010). Bone metastasis means tumor progression. Hypoproteinemia is prevalent in cancer patients due to the damage inflicted on the body by the tumor and various treatment methods (Christina et al. 2023; Jiang et al. 2022; Sun et al. 2022); therefore, these patients require a higher protein intake to maintain body functions (Muscaritoli et al. 2021). Adequate plasma albumin has been demonstrated in many studies to be the basis for improved prognosis in patients with various medical conditions (Fanali et al. 2012; Yu et al. 1877; Amouzandeh et al. 2018; Arques 2018). Meanwhile, a prospective cohort study showed a significant negative correlation between serum albumin and the inflammatory marker C-reactive protein (Sheinenzon et al. 2021). Serum chloride ions are important electrolytes for maintaining body fluid homeostasis and are associated with the cardiac, renal and neurohormonal systems (Zandijk et al. 2021). Chloride was associated with acidosis and we included acidosis for analysis, but acidosis did not show a correlation with patient prognosis. The effect of serum chloride ions on patient prognosis is controversial to some extent. A study by Yaling Zhai et al. showed that elevated serum chloride levels were associated with poor prognostic outcomes in patients with IgA nephropathy, which contradicts our findings (Zhai et al. 2021). Nevertheless, some studies have shown that electrolyte disorders such as hypochlorhydria are significantly associated with poor prognosis in cancer patients (Li et al. 2020). In addition, a study on heart failure revealed a significant inverse association between serum chloride concentration and long-term mortality of patients (HR: 0.890; 95% CI: 0.863–0.918; *P* < 0.001), which is consistent with our study. Therefore, we believe that the effect of serum chloride ions on patient mortality is related to the disease characteristics of patients. However, no studies have directly illustrated the effect of serum chloride concentration on the prognosis of patients with bone metastases, and more research data are needed for validation. Among the biomarkers that manifest as mortality-promoting factors, lack of oxygen in the body affects the normal function of many organs (Fenves and Emmett 2021). In our model, the biomarkers incorporated into the model are mostly indicators reflecting acid–base and electrolyte balance and nutritional status in the patient’s body. Hence, for patients with secondary bone tumors in the intensive care unit, the administration of adequate nutrients and maintenance of acid-base balance are important measures to improve the prognosis of patients. In addition, hepatic metastases and the prognostic score were also major risk factors. This suggests that the severity of the cancer and the patient's physical condition are equally significant in predicting prognosis.

Our model can provide valid predictive information, but some limitations need to be mentioned: first, due to the limitation of the database, we could not include some important indicators, such as the primary tumor of the patient, the size of the primary tumor, and the site of metastasis. Second, we were unable to determine whether the patient’s combined tumor was the primary tumor. Moreover, some laboratory indicators may interact with each other, but we are unable to detect the interactions between covariates. Finally, our model only used data from a single center and needs to be validated using a large sample of data from multiple centers.

## Conclusion

A prognostic model has been developed in this study for patients with secondary bone tumors in the intensive care unit. The prediction performance of the model is robust and it can provide valid forecasting information. The indicators included in the model suggest that nutritional support and maintenance of fluid balance are important therapeutic measures to improve the prognosis of patients with bone metastases in the intensive care unit.

## Data Availability

All data generated or analyzed during this study are included in this published article or are available from the corresponding author on reasonable request.
